# Machine Learning–Based Prediction of Neurodegenerative Disease in Patients With Type 2 Diabetes by Derivation and Validation in 2 Independent Korean Cohorts: Model Development and Validation Study

**DOI:** 10.2196/56922

**Published:** 2024-10-03

**Authors:** Hyunji Sang, Hojae Lee, Jaeyu Park, Sunyoung Kim, Ho Geol Woo, Ai Koyanagi, Lee Smith, Sihoon Lee, You-Cheol Hwang, Tae Sun Park, Hyunjung Lim, Dong Keon Yon, Sang Youl Rhee

**Affiliations:** 1 Department of Endocrinology and Metabolism Kyung Hee University Medical Center, Kyung Hee University College of Medicine Seoul Republic of Korea; 2 Center for Digital Health, Medical Science Research Institute Kyung Hee University Medical Center, Kyung Hee University College of Medicine Seoul Republic of Korea; 3 Department of Regulatory Science Kyung Hee University Seoul Republic of Korea; 4 Department of Family Medicine Kyung Hee University Medical Center, Kyung Hee University College of Medicine Seoul Republic of Korea; 5 Department of Neurology Kyung Hee University Medical Center, Kyung Hee University College of Medicine Seoul Republic of Korea; 6 Research and Development Unit Parc Sanitari Sant Joan de Deu Barcelona Spain; 7 Centre for Health, Performance and Wellbeing Anglia Ruskin University Cambridge United Kingdom; 8 Department of Internal Medicine Gachon University College of Medicine Incheon Republic of Korea; 9 Division of Endocrinology and Metabolism, Department of Internal Medicine Kyung Hee University Hospital at Gangdong and Kyung Hee University School of Medicine Seoul Republic of Korea; 10 Division of Endocrinology and Metabolism, Department of Internal Medicine, Research Institute of Clinical Medicine Jeonbuk National University, Jeonbuk National University Medical School Jeonju Republic of Korea; 11 Department of Medical Nutrition, Graduate School of East-West Medical Science Kyung Hee University Yongin Republic of Korea; 12 Department of Precision Medicine Kyung Hee University College of Medicine Seoul Republic of Korea; 13 Department of Pediatrics Kyung Hee University College of Medicine Seoul Republic of Korea

**Keywords:** machine learning, neurodegenerative disease, diabetes mellitus, prediction, AdaBoost

## Abstract

**Background:**

Several machine learning (ML) prediction models for neurodegenerative diseases (NDs) in type 2 diabetes mellitus (T2DM) have recently been developed. However, the predictive power of these models is limited by the lack of multiple risk factors.

**Objective:**

This study aimed to assess the validity and use of an ML model for predicting the 3-year incidence of ND in patients with T2DM.

**Methods:**

We used data from 2 independent cohorts—the discovery cohort (1 hospital; n=22,311) and the validation cohort (2 hospitals; n=2915)—to predict ND. The outcome of interest was the presence or absence of ND at 3 years. We selected different ML-based models with hyperparameter tuning in the discovery cohort and conducted an area under the receiver operating characteristic curve (AUROC) analysis in the validation cohort.

**Results:**

The study dataset included 22,311 (discovery) and 2915 (validation) patients with T2DM recruited between 2008 and 2022. ND was observed in 133 (0.6%) and 15 patients (0.5%) in the discovery and validation cohorts, respectively. The AdaBoost model had a mean AUROC of 0.82 (95% CI 0.79-0.85) in the discovery dataset. When this result was applied to the validation dataset, the AdaBoost model exhibited the best performance among the models, with an AUROC of 0.83 (accuracy of 78.6%, sensitivity of 78.6%, specificity of 78.6%, and balanced accuracy of 78.6%). The most influential factors in the AdaBoost model were age and cardiovascular disease.

**Conclusions:**

This study shows the use and feasibility of ML for assessing the incidence of ND in patients with T2DM and suggests its potential for use in screening patients. Further international studies are required to validate these findings.

## Introduction

Neurodegenerative diseases (NDs) are characterized by the progressive dysfunction of synapses, neurons, glial cells, and their networks [1]. NDs include dementia, Parkinson disease (PD), multiple sclerosis, Huntington disease, and amyotrophic lateral sclerosis [1]. Risk factors for dementia, the most common type of ND, include older age, genetic risk factors (family history of dementia and Apolipoprotein E), cardiometabolic risk factors (diabetes mellitus, hypertension, dyslipidemia, obesity, and vascular disease), smoking, hearing impairment, depression, less education, physical inactivity, alcohol consumption, traumatic brain injury, and air pollution [2-7]. Known risk factors for Parkinson disease, the second most common ND, include advanced age, male sex, family history of Parkinson disease, environmental exposure (pesticides and air pollution), and comorbidities (obesity, metabolic syndrome, diabetes mellitus, traumatic brain injury, and a history of melanoma or prostate cancer) [8-13].

Type 2 diabetes mellitus (T2DM) is a significant health problem and it requires careful management because it can be accompanied by several complications. In addition to well-known diabetic complications such as retinopathy, neuropathy, and nephropathy, T2DM plays a vital role in the development of cardiovascular, peripheral vascular, and cerebrovascular diseases [14]. Recently, the link between T2DM and the development of NDs has gained attention [15]. The incidence of dementia and Alzheimer disease (AD) among people with diabetes is estimated to be 9.5 and 6.8 per 1000 person-years, respectively [16]. Parkinson disease has affected 31,577 people with T2DM as of 2016 [17]. Compared to healthy people, people with impaired fasting glucose and diabetes for less than 5 years and diabetes for more than 5 years have a 1.04-fold, 1.19-fold, and 1.62-fold higher risk of Parkinson disease, respectively [17].

In clinical practice, biomarkers are needed to accurately diagnose NDs and identify their underlying pathogenesis [18,19]. Current guidelines for ND prevention strategies state that prevention should be based on a plan to reduce modifiable risk factors [20]. To effectively prevent ND in primary care, where most cognitively impaired patients with suspected ND are observed, it is essential to identify good predictors of ND among the standard physical examinations and laboratory tests performed for health screening purposes. To date, there is a lack of information on the correlation and dominance of these predictors.

Recent developments in artificial intelligence have focused on applying novel techniques, such as machine learning (ML), to existing disease models [21]. ML is a powerful tool that can overcome existing limitations using clinical data to uncover hidden patterns and identify critical variables associated with disease development. Clinicians can efficiently detect early warning signs and risk factors of complications by integrating ML algorithms with clinical data [22]. Recently, several models using ML have been developed to predict ND in T2DM [23-25]. The purpose of this study was to identify the relationship between clinical factors and ND and to develop a predictive model for the occurrence of ND through intensive model training and validation by taking advantage of the strengths of ML technology in patients with T2DM in South Korea.

## Methods

### Study Population and Data Collection

**Figure 1:**
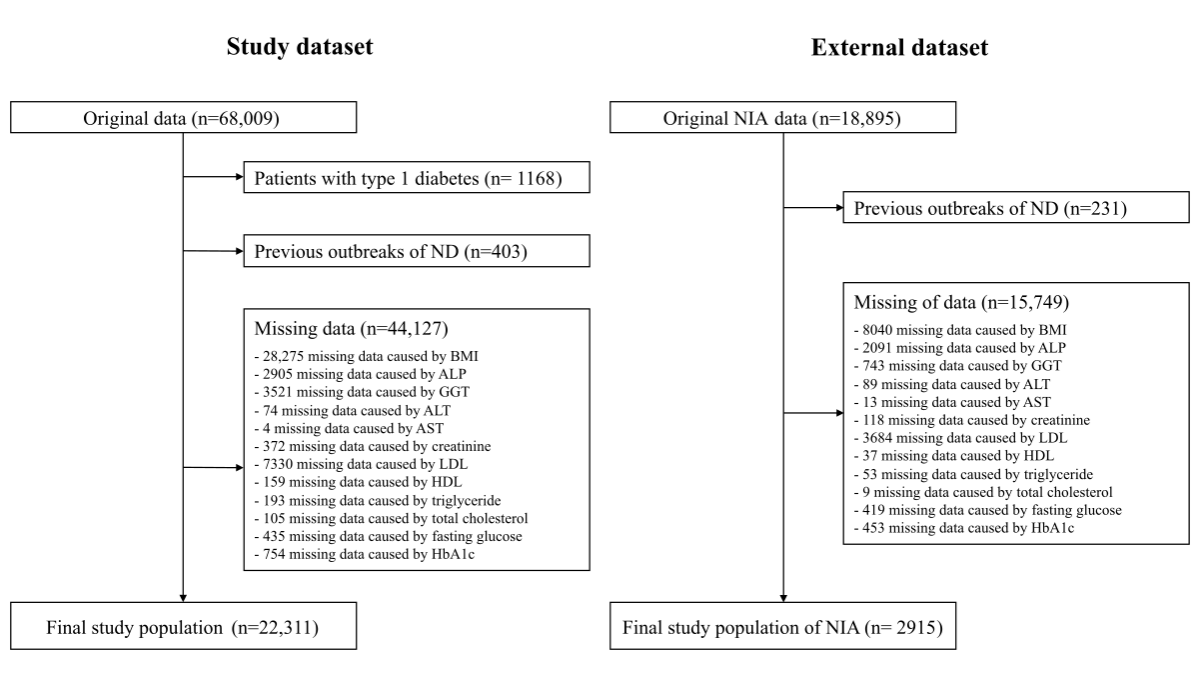
Study workflow. ALP: alkaline phosphatase; ALT: alanine aminotransferase; AST: aspartate aminotransferase; GGT: gamma-glutamyl transferase; HbA1c: glycated hemoglobin; HDL: high-density lipoprotein; LDL: low-density lipoprotein; ND: neurodegenerative disease; NIA: National Information Society Agency.

This retrospective study used data from 2 independent longitudinal cohorts previously enrolled in an observational study. Hospital-based data were collected from January 1, 2008, to December 31, 2022. Eligible participants were selected from patients with T2DM, and those with type 1 diabetes or a history of ND were excluded. Finally, 22,311 patients from a tertiary hospital at the Kyung Hee University Medical Center were selected as the discovery cohort. Data for extra validation were collected from a retrospective dataset from the secondary hospitals, Kyung Hee University Medical Center at Gangdong and Gachon University Gil Hospital (validation cohort), and 2915 eligible patients were selected (Figure 1).

### Input Variables

A comprehensive set of 56 variables is included in the model. Baseline patient demographics include age and sex, and medical histories include the presence of hypertension, dyslipidemia, macrovascular complications (cardiovascular and peripheral vascular diseases), microvascular complications (retinopathy, chronic kidney disease, end-stage renal disease, and neuropathy), and cancer. Medication history includes types of antidiabetic agents (metformin, sulfonylurea, dipeptidyl peptidase-4 inhibitor, meglitinide, thiazolidinedione, α-glucosidase inhibitor, insulin, glucagon-like peptide-1 receptor agonist, and sodium-glucose co-transporter 2 inhibitor), antihypertensive drugs (angiotensin II receptor blocker [ARB], angiotensin-converting enzyme inhibitor [ACEi], calcium channel blocker [CCB], diuretics, and beta-blocker), dyslipidemia drugs (statin, fibrate, and ezetimibe), and antiplatelet agents (aspirin, clopidogrel, cilostazol, and glycoprotein IIb/IIIa antagonist). The clinical parameters include BMI [26]. Blood tests included those for glycated hemoglobin (HbA_1c_), serum glucose, total cholesterol, triglyceride, high-density lipoprotein (HDL), low-density lipoprotein (LDL), serum creatinine, aspartate aminotransferase (AST), alanine aminotransferase (ALT), gamma-glutamyl transferase (GGT), and alkaline phosphatase (ALP). For BMI and blood test results, we used the median and standard deviation of each parameter as input variables.

### Identification of New ND Cases

New-onset ND in patients with T2DM was identified using the *ICD-10* (*International Statistical Classification of Diseases, Tenth Revision*) codes for dementia (F00.X–F03.X and G30.X) and Parkinson disease (G20.X). The primary end point was new-onset ND within 3 years.

### Data Preprocessing

Missing data were excluded from the analysis. The covariates were divided into three sections: (1) demographics, (2) physical examination and blood tests, and (3) medication and comorbidities. Using the examination date, we used the physical examination and blood test data before the ND outbreak. The dataset was calculated for the entire study period and converted to a mean value before the onset of ND. Information on medications and comorbidities at the first visit was used as a covariate. Data are presented as number (%) or mean (SD).

### Model Training and Validation

A common ML approach for prediction involves dividing data into training and test sets. In this study, the target value of the given data on the incidence of ND over 3 years was insufficient. Therefore, the model was trained on the entire dataset rather than splitting it for internal validation. Although including a separate test set is beneficial for providing an unbiased evaluation of the model performance on unseen data from the same distribution as the training data, using an external dataset for validation has its own merits. Validating the model with data from different distributions can verify its robustness and applicability in various real-world settings. A separate external dataset was used to assess the extent to which the model would have generalizability. This approach is essential for verifying whether the model performs well on new and previously unseen data.

### Model Development

We chose decision tree–based ensemble models, such as AdaBoost, LightGBM, Random Forest, and XGBoost. Hyperparameter tuning was performed using GridSearchCV, and the area under the receiver operating characteristic curve (AUROC) was maximized to determine the best combination of hyperparameters for optimizing the performance of each model.

### ML Analysis

Various tree-based and linear classification models have been used to determine AUROC scores for predicting ND occurrence. GridSearchCV was used to optimize the hyperparameters of models, using the AUROC score as a scoring metric. After determining the optimal hyperparameters, the model is trained for subsequent predictions. Given the class imbalance in our data, we used a synthetic minority oversampling technique to generate synthetic samples. We used various metrics, such as AUROC, accuracy, sensitivity, specificity, and balanced accuracy, to evaluate the model's performance. These metrics are calculated based on the probability predictions produced by the model. A 10-fold stratified cross-validation was performed to assess the model’s ability to handle new data. The Youden index was used for each stratification to identify the optimal threshold [27]. Subsequently, we calculated the mean and 95% CIs for each performance metric to measure the average and variability of the model's performance.

A receiver operating characteristic (ROC) curve was plotted to illustrate the performance of the model. This was complemented by the mean ROC curve and SD within that range, demonstrating the distribution of the model performance. Because the AdaBoost model yielded the highest AUROC score among the various decision tree models tested, we selected this model to identify the most important features for predicting ND. The importance of each feature was extracted using the feature importance attributes of the AdaBoost model. We selected the top 15 features with the largest impact on the model and plotted them on a bar graph to visualize their influence on the model predictions. Logistic regression analysis was used to obtain odds ratios (ORs) to compare the effects of the different medications on ND occurrence.

### Performance Metrics

To comprehensively understand the performance of our model, we selected 5 performance metrics: AUROC, accuracy, sensitivity, specificity, and balanced accuracy. The AUROC is a robust performance measure that assesses a model’s ability to discriminate between classes across all possible thresholds. Its robustness originates from the fact that it considers both sensitivity and specificity, making it a preferred metric, particularly in situations where classes are imbalanced. Accuracy is a simple and intuitive performance metric that indicates the proportion of true results (both true positives and true negatives) from the total number of cases examined. However, accuracy alone can be misleading, particularly for unbalanced datasets; therefore, additional performance metrics are required. Sensitivity and specificity were used to assess how well the model identified the positive and negative cases, respectively. Sensitivity provides insight into a model’s ability to detect positive cases by measuring the proportion of true positives correctly identified by the model. Specificity is a measure of the proportion of true negatives that are correctly identified and provides a sense of a model’s ability to avoid false alarms. Finally, we include balanced accuracy to provide a more balanced view of the performance of our model, particularly in the face of class disparity. As an average of sensitivity and specificity, balanced accuracy assigns equal weights to both metrics, making it an excellent alternative to accuracy when addressing unbalanced datasets. Combining these metrics enables us to evaluate the performance of our model from different perspectives, thereby ensuring a more robust evaluation [28,29].

### Software and Libraries

Data preprocessing, model development, and analyses were conducted using Python (version 3.9.16; Python Software Foundation). The main libraries used in our study include Scikit-learn 1.2.2, NumPy 1.23.5, and Pandas 1.5.3 for ML algorithms and data manipulation. Matplotlib 3.7.1 and Seaborn 0.12.2 were used for data visualization.

### Ethical Considerations

This study was approved by the Institutional Review Board of the Kyung Hee University Hospital (KHSIRB-22-473(EA)). The requirement for informed consent was waived by the institutional review board because de-identified data were used in the analyses. This study followed the guidelines outlined in the TRIPOD (Transparent Reporting of a Multivariate Prediction Model for Individual Prognosis or Diagnosis) statement. Participants in this study did not receive compensation as the data were anonymized.

## Results

### Cohort Characteristics

A total of 22,311 patients were selected from the discovery cohort, of whom 133 (0.6%) had ND. Among the participants, 11,545 (51.8%) were male, and the mean age was 63.5 (SD 12.0) years. For additional validation, 2915 patients were included, including 15 (0.5%) patients with ND from the validation cohort. The validation cohort had 1625 (55.8%) men with a mean age of 57.8 (SD 11.8) years (Table 1).

**Table 1:** Baseline characteristics of the discovery and validation datasets.

							Discovery dataset										Validation dataset						
							Total (N=22,311)			Control (N=22,178)			Case^a^ (N=133)			Total (N=2915)				Control (N=2900)			Case^a^ (N=15)
Age, mean (SD)							63.5 (12.0)			63.4 (11.9)			73.3 (7.6)			57.8 (11.8)				57.8 (11.8)			69.5 (10.6)
Male, n (%)							11,545 (51.8)			11,492 (51.8)			53 (39.9)			1625 (55.8)				1617 (55.8)			8 (53.3)
BMI, kg/m^2^, mean (SD)							24.9 (3.6)			24.9 (3.6)			24.1 (3.6)			25.2 (3.4)				25.2 (3.4)			23.3 (2.7)
**Blood test, mean (SD)**																							
	HbA_1c_^b^, (%)					6.78 (1.00)			6.78 (1.00)			6.77 (0.84)			7.19 (1.14)				7.19 (1.14)			7.64 (1.37)	
	Fasting blood glucose, mg/dL					146.8 (44.9)			146.8 (44.9)			149.0 (45.9)			145.9 (45.7)				145.8 (45.7)			155.8 (38.5)	
	Total cholesterol, mg/dL					157.9 (33.9)			157.9 (34.0)			155.1 (28.3)			163.1 (33.0)				163.2 (32.9)			142.2 (34.4)	
	Triglyceride, mg/dL					141.8 (67.9)			141.9 (68.0)			129.9 (51.0)			147.8 (67.9)				147.7 (67.8)			148.9 (86.0)	
	HDL^c^ cholesterol, mg/dL					47.4 (11.9)			47.4 (11.9)			48.0 (11.6)			45.2 (10.0)				45.3 (10.0)			37.6 (10.7)	
	LDL^d^ cholesterol, mg/dL					90.3 (27.1)			90.4 (27.1)			87.6 (20.8)			92.5 (29.8)				92.6 (29.8)			76.9 (35.8)	
	Creatinine, mg/dL					0.90 (0.44)			0.90 (0.44)			0.96 (0.48)			1.08 (0.89)				1.08 (0.89)			1.42 (1.28)	
	AST^e^, U/L					26.9 (13.5)			26.9 (13.5)			25.1 (9.4)			24.7 (8.4)				24.7 (8.4)			22.6 (9.2)	
	ALT^f^, U/L					24.5 (14.2)			24.5 (14.2)			19.6 (9.6)			25.6 (13.2)				25.7 (13.2)			15.4 (6.7)	
	GGT^g^, U/L					38.3 (35.8)			38.4 (35.8)			33.0 (30.4)			38.4 (32.4)				38.4 (32.5)			31.3 (18.6)	
	ALP^h^, U/L					79.1 (25.6)			79.1 (25.6)			75.3 (21.9)			171.6 (89.0)				172.1 (89.0)			70.8 (23.3)	
**Comorbid conditions, n (%)**																							
	Hypertension					9440 (42.3)			9,381 (42.3)			59 (44.4)			1670 (57.3)				1660 (57.2)			10 (66.7)	
	Dyslipidemia					10,389 (46.6)			10,334 (46.6)			55 (41.4)			1419 (48.7)				1418 (48.9)			1 (6.7)	
**Macrovascular complications, n (%)**																							
	Cardiovascular disease^i^					8310 (37.3)			8257 (37.2)			53 (39.9)			980 (33.6)				968 (33.4)			12 (80.0)	
	Peripheral vascular disease					129 (0.6)			129 (0.6)			N/A			422 (14.5)				421 (14.5)			1 (6.7)	
**Microvascular complications, n (%)**																							
	Retinopathy					1670 (7.5)			1664 (7.5)			6 (4.5)			488 (16.7)				487 (16.8)			1 (6.7)	
	Chronic kidney disease					2138 (9.6)			2131 (9.6)			7 (5.3)			582 (20.0)				580 (20.0)			2 (13.3)	
	ESRD^j^					145 (0.7)			144 (0.7)			1 (0.8)			178 (6.1)				176 (6.1)			2 (13.3)	
	Neuropathy					5061 (22.7)			5027 (22.7)			34 (25.6)			700 (24.0)				698 (24.1)			2 (13.3)	
	Cancer					3609 (16.2)			3604 (16.3)			5 (3.8)			303 (10.4)				302 (10.4)			1 (6.7)	
**Medication use, n (%)**																							
	**Diabetes mellitus**																						
				Metformin	12,300 (55.1)			12,227 (55.1)			73 (54.9)			1219 (41.8)				1219 (42.0)			N/A		
				Sulfonylurea	7188 (32.2)			7135 (32.2)			53 (39.9)			462 (15.9)				462 (15.9)			N/A		
				DPP-4^k^ inhibitor	5374 (24.1)			5347 (24.1)			27 (20.3)			195 (6.7)				195 (6.7)			N/A		
				Meglitinide	985 (4.4)			972 (4.4)			13 (9.8)			171 (5.9)				171 (5.9)			N/A		
				Thiazolidinedione	1,367 (6.1)			1,356 (6.1)			11 (8.3)			40 (1.4)				40 (1.4)			N/A		
				α-Glucosidase inhibitor	1,007 (4.5)			1,001 (4.5)			6 (4.5)			161 (5.5)				161 (5.6)			N/A		
				Insulin	7,188 (32.2)			7,135 (32.2)			53 (39.9)			N/A				N/A			N/A		
				GLP-1^l^ receptor agonist	32 (0.1)			32 (0.1)			N/A			N/A				N/A			N/A		
				SGLT2^m^ inhibitor	634 (2.8)			631 (2.9)			3 (2.3)			N/A				N/A			N/A		
	**Hypertension**																						
				Angiotensin II receptor blocker	10,063 (45.1)			9999 (45.1)			64 (48.1)			245 (8.4)				245 (8.5)			N/A		
				ACE inhibitor	1735 (7.8)			1724 (7.8)			11 (8.3)			125 (4.3)				125 (4.3)			N/A		
				Calcium channel blocker	10,485 (47.0)			10,420 (47.0)			65 (48.9)			603 (20.7)				603 (20.8)			N/A		
				Diuretics	7280 (32.6)			7228 (32.6)			52 (39.1)			229 (7.9)				229 (7.9)			N/A		
				Beta blocker	5981 (26.8)			5936 (26.8)			45 (33.8)			2 (0.1)				2 (0.1)			N/A		
	**Dyslipidemia**																						
			Statin			12,711 (57.0)			12,625 (56.9)			86 (64.7)			958 (32.9)				958 (33.0)			N/A	
			Fibrate			896 (4.0)			892 (4.0)			4 (3.0)			92 (3.2)				92 (3.2)			N/A	
			Ezetimibe			1542 (6.9)			1531 (6.9)			11 (8.3)			106 (3.6)				106 (3.7)			N/A	
	**Antiplatelet**																						
		Aspirin				9136 (41.0)			9064 (40.9)			72 (54.1)			762 (26.1)				762 (26.3)			N/A	
		Clopidogrel				6406 (28.7)			6359 (28.7)			47 (35.3)			309 (10.6)				309 (10.7)			N/A	
		Cilostazol				2707 (12.1)			2682 (12.1)			25 (18.8)			164 (5.6)				164 (5.7)			N/A	
		Glycoprotein IIb/IIIa antagonist				279 (1.3)			276 (1.2)			3 (2.3)			N/A				N/A			N/A	

^a^Group of patients with newly developed neurodegenerative disease within 3 years.

^b^HbA_1c_: glycated hemoglobin.

^c^HDL: high-density lipoprotein.

^d^LDL: low-density lipoprotein.

^e^AST: aspartate transaminase.

^f^ALT: alanine transaminase.

^g^GGT: gamma-glutamyl transferase.

^h^ALP: alkaline phosphatase.

^i^Cardiovascular diseases included ischemic heart disease, myocardial infarction, heart failure, atrial fibrillation, stroke, and other cerebrovascular diseases.

^j^ESRD: end-stage renal disease.

^k^DPP-4: dipeptidyl peptidase-4.

^l^GLP-1: glucagon-like peptide-1.

^m^SGLT2: sodium-glucose co-transporter 2.

### Comparisons of Prediction Model Performance

The AdaBoost model performed well on the discovery set (AUROC 0.82, 95% CI 0.79-0.85; accuracy 74.2%, 95% CI 70.9-77.4; sensitivity 73.6%, 95% CI 69.9-77.3; specificity 74.2%, 95% CI 70.9-77.4; and balanced accuracy 73.9%, 95% CI 70.6-77.2). The LightGBM model performed next best (AUROC 0.791, 95% CI 0.756-0.825; accuracy 72.5%, 95% CI 68.1-76.8; sensitivity 71.8%, 95% CI 67.0-76.6; specificity 72.5%, 95% CI 68.1-76.8; and balanced accuracy 72.2%, 95% CI 68.0-76.3). The Random Forest model closely followed, yielding an AUROC of 0.79 (95% CI 0.76-0.82) and solid metrics (accuracy 69.3%, 95% CI 63.0-75.5; sensitivity 69.1%, 95% CI 63.6-74.6; specificity 69.3%, 95% CI 63.0-75.6; and balanced accuracy 69.2%, 95% CI 63.4-75.0). The XGBoost model also had similar performance (AUROC 0.79, 95% CI 0.77-0.81; accuracy 72.0%, 95% CI 68.5-75.6; sensitivity 69.1%, 95% CI 65.7-72.5; specificity 72.0%, 95% CI 68.5-75.6; and balanced accuracy 70.6%, 95% CI 67.4-73.8; Table 2).

**Table 2:** Performance metrics of 4 different ML^a^ algorithms on the original and external validation datasets.

Model			AUROC^b^		Accuracy, (%)		Sensitivity, (%)		Specificity, (%)		Balanced accuracy, (%)
**Original dataset**											
	ADB^c^	0.819 (0.786–0.851)		74.2 (70.9-77.4)		73.6 (69.9-77.3)		74.2 (70.9-77.4)		73.9 (70.6-77.2)	
	LGB^d^	0.791 (0.756-0.825)		72.5 (68.1-76.8)		71.8 (67.0-76.6)		72.5 (68.1-76.8)		72.2 (68.0-76.3)	
	RF^e^	0.788 (0.757-0.819)		69.3 (63.0-75.5)		69.1 (63.6-74.6)		69.3 (63.0-75.6)		69.2 (63.4-75.0)	
	XGB^f^	0.788 (0.766-0.810)		72.0 (68.5-75.6)		69.1 (65.7-72.5)		72.0 (68.5-75.6)		70.6 (67.4-73.8)	
**Validation dataset**											
	ADB	0.830		78.6		78.6		78.6		78.6	
	LGB	0.833		84.3		78.6		84.4		81.5	
	RF	0.820		77.0		78.6		76.9		77.8	
	XGB	0.786		74.3		71.4		74.3		72.9	

^a^ML: machine learning.

^b^AUROC: area under the receiver operating characteristic curve.

^c^ADB: AdaBoost.

^d^LGB: LightGBM.

^e^RF: Random Forest.

^f^XGB: XGBoost.

Performance metrics of 4 machine learning models (AdaBoost, LightGBM, Random Forest, and XGBoost) were provided for the prediction of the onset of neurodegenerative disease within 3 years in patients, using both the original and an additional external validation dataset.

Upon applying these models to the external validation set, the AdaBoost and LightGBM models achieved high AUROCs of 0.83 and 0.83, respectively. The Random Forest and XGBoost models exhibited improved performance metrics with AUROC values of 0.82 and 0.79, respectively (Table 2).

**Figure 2:**
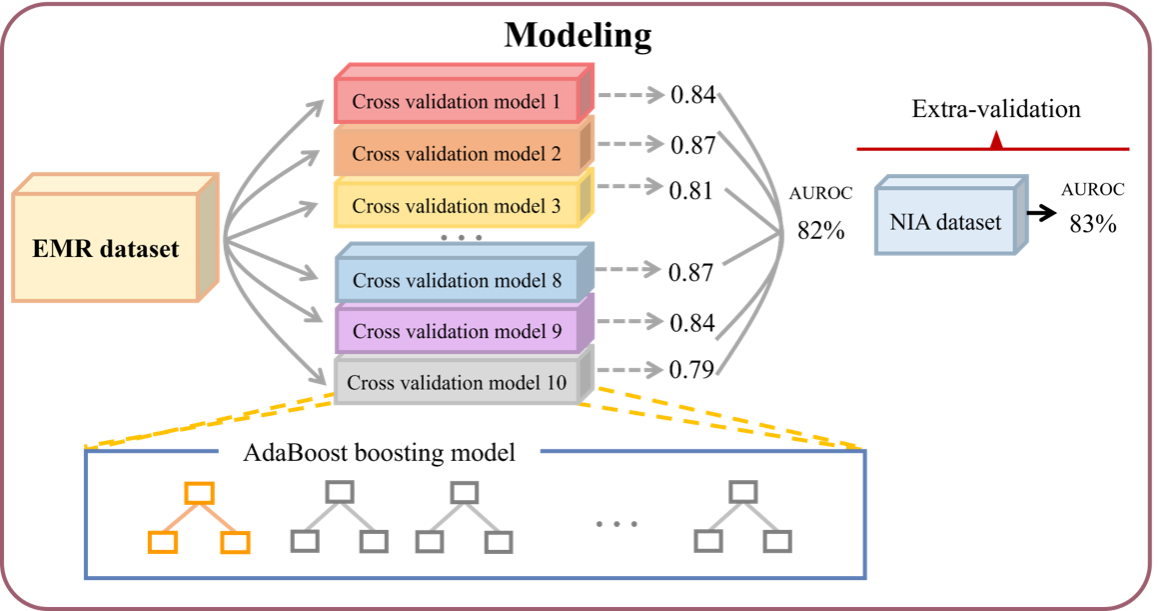
Model architecture. An electronic medical record (EMR) dataset from Kyung Hee University Medical Center was used for model development processed by 10-fold cross-validation and AdaBoost. Extravalidation was executed by the National Information Society Agency (NIA) dataset from Kyung Hee University Hospital at Gangdong and Gachon University Gil Medical Center.

**Figure 3:**
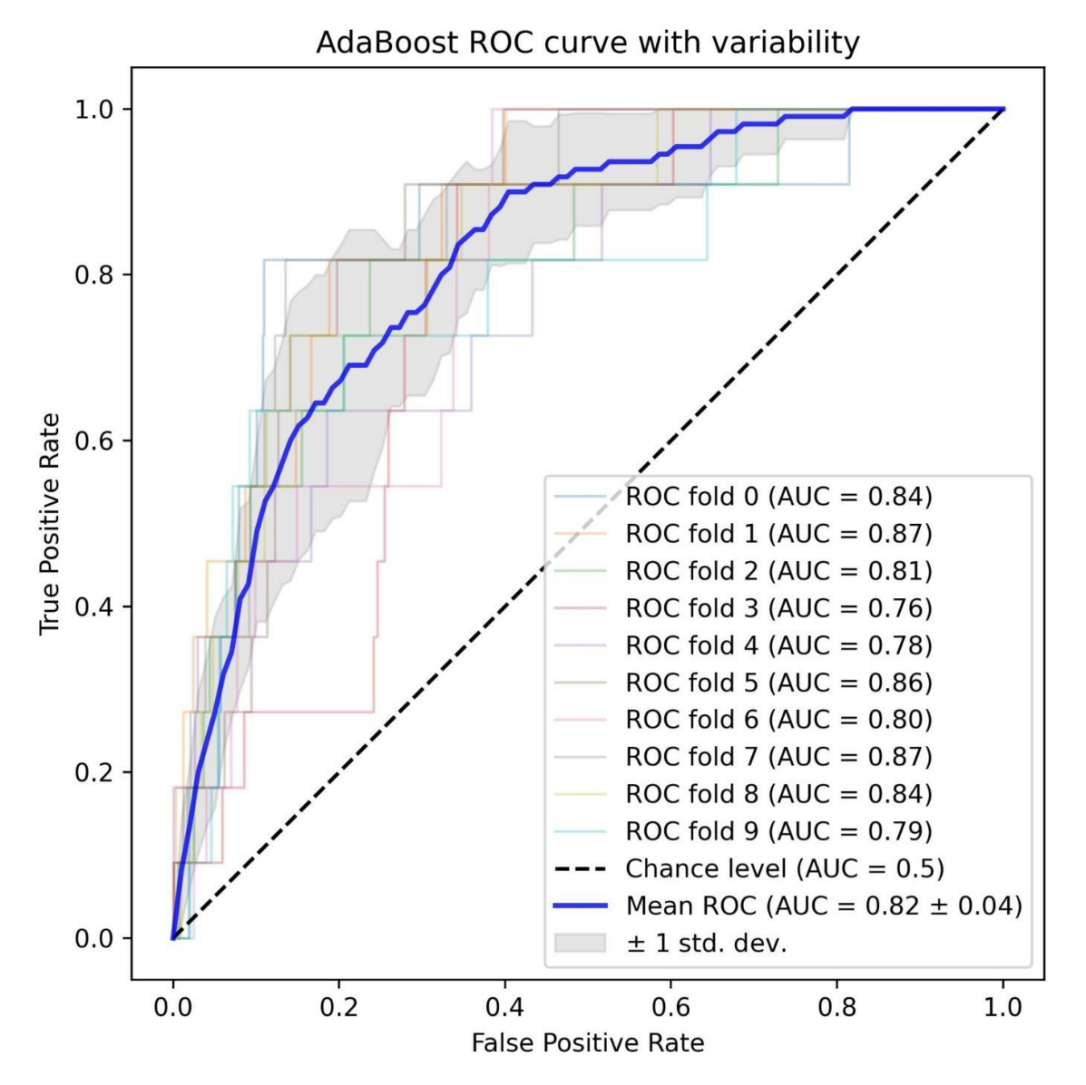
ROC curves of the AdaBoost model. Mean ROC curve from 10-fold cross-validation on the original dataset. AUC: area under the receiver operating characteristic curve; ROC: receiver operating characteristic.

Consequently, with excellent and consistent results in both independent datasets, the AdaBoost model emerged as the best predictor of ND development within 3 years among patients with diabetes (Figures 2 and 3).

### Feature Importance for Improving Interpretability of ML Models

**Figure 4:**
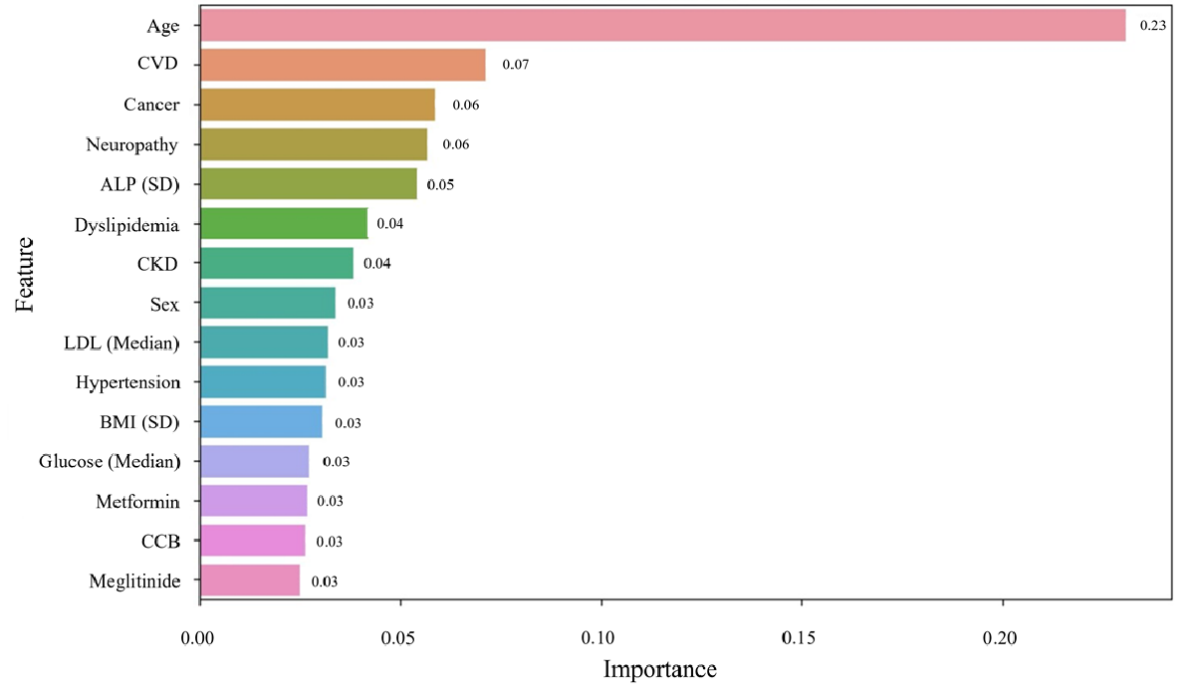
Top 15 feature-importance of AdaBoost model. ALP: alkaline phosphatase; CCB: calcium channel blocker; CKD: chronic kidney disease; CVD: cardiovascular disease; LDL: low-density lipoprotein.

The impacts of the contributing factors analyzed using the feature importance method are shown in Figure 4. Among the 56 variables considered in this study, age was the most important factor that contributed to the performance of the ND prediction model, followed by cardiovascular disease, cancer, neuropathy, and ALP levels. For other comorbidities, dyslipidemia, chronic kidney disease, and hypertension were among the top 10 feature importance, and for medications, metformin, calcium channel blockers, and meglitinide were among the top 15 feature importance.

### Comparison of the Impact of Different Drugs on ND Occurrence

We compared the risk of developing ND according to the type of medication previously administered by the participants by calculating ORs. Among antidiabetic drugs and antiplatelet drugs, meglitinide (OR 0.42, 95% CI 0.24-0.75), aspirin (OR 0.59, 95% CI 0.42-0.82), and cilostazol (OR 0.59, 95% CI 0.38-0.92) were associated with a significantly reduced risk of ND. The remaining antidiabetic, antihypertensive, antihyperlipidemic, and antiplatelet agents did not significantly increase or decrease the risk of ND development (Multimedia Appendix 1).

## Discussion

This study emphasized the importance of developing a highly accurate ML-based ND prediction model that can be universally applied to adults with T2DM in South Korea. This study provided a simple and precise assessment of the future annual risk of ND in people with diabetes nationwide. The AdaBoost, LightGBM, Random Forest, and XGBoost ensemble models showed excellent performance with AUROC values ranging from 0.79 to 0.82 on the discovery dataset and 0.79 to 0.83 on the external validation dataset. Age and cardiovascular disease were the top 15 factors affecting the feature importance. The results of this study can potentially improve patient outcomes by enabling timely intervention, advancing the comprehension of contributing variables, and reducing the burden of neurodegenerative complications in patients with T2DM.

This study was based on a large cohort of the Korean population and used data from 3 university hospitals. Multiple variables, such as anthropometric variables, medical history, medication use, and laboratory tests, were used for model development. An advantage of this study is that long-term follow-up data of approximately 3 years were available for outcome evaluation. This ND prediction model is meaningful because it demonstrates sufficiently good performance, with a mean AUROC of 0.82, using only questionnaires, body measurements, and blood tests commonly conducted in clinical practice for patients with diabetes.

Our findings provide insights into the metrics that can be used in primary care for ND prediction. In addition to the risk factors considered by our ML model, other known risk factors for the development of ND include genetic risk factors, lifestyle factors, environmental exposure, and traumatic brain injury. Existing biomarkers for ND are related to amyloid beta or tau proteins, which are involved in the pathophysiology of ND and are measured using neuroimaging techniques, such as brain magnetic resonance imaging, single-photon emission computed tomography or positron emission tomography, or cerebrospinal fluid testing [30,31]. Additionally, biomarkers using blood samples exist, such as high-sensitivity C-reactive protein, GGT, homocysteine, apolipoprotein E, and uric acid [30,31]. Although they can be used as adjuncts to increase diagnostic confidence, most are expensive or invasive and are not recommended as routine diagnostic tests in clinical practice. As we aimed to predict ND risk for screening purposes in primary health care centers, we focused on constructing an ML model that can predict ND risk based on general physical measurements and blood tests without requiring specialized tests.

In this study, we performed a feature-importance analysis on the interpretability of the AdaBoost model, which performed the best among the models investigated. According to feature importance analysis, age, cardiovascular disease, cancer, neuropathy, and ALP levels were among the top 5 predictors of ND. The association between age, cardiovascular disease, and neuropathy with ND was consistent with the results of previous studies. Age is a conventional risk factor for ND [32]. Cardiovascular disease is a known risk factor for ND [33]. Cardiometabolic risk factors such as diabetes, hypertension, and hyperlipidemia were also consistently associated with the risk of developing ND [34]. The association between peripheral neuropathy and ND in this study is consistent with its association with the development of mild cognitive impairment and dementia in the general population and people with diabetes [35]. Meanwhile, the relationship between cancer and ND is likely to be inverse according to previous studies. The incidence of cancer is reportedly lower in patients with ND [36]. It is important to note that aging also affects the occurrence of cancer [37], and this study did not adjust for the effect of aging on cancer; therefore, further research is needed to determine the causality between cancer itself and ND. The results related to ALP levels were consistent with previous reports showing that ALP levels were increased in patients with AD [38]. In contrast, some studies have found no significant association between ALP and PD [39]. This may be related to increased bone ALP, as PD is associated with an increased incidence of osteoporosis, falls, and fractures [40]. Moreover, the association between ALP variability and ND development has not been previously studied and warrants further investigation.

Although the AdaBoost model identified important features, such as the use of metformin, CCB, and meglitinide, the results of logistic regression analysis showed that only meglitinide significantly reduced the risk of ND. This difference arises because AdaBoost can capture complex patterns and nonlinear interactions among variables that logistic regression may not fully capture because of its linear assumptions. The ability of AdaBoost to highlight nonlinear relationships provides additional insights into the factors affecting ND risk.

While some studies have shown an increased incidence of ND with long-term exposure to metformin [41], conflicting studies have suggested that metformin has a therapeutic potential for ND [42]. Because metformin users may have more hyperglycemia than nonusers, it is difficult to conclude that metformin use worsens the risk of developing ND. However, few studies have investigated the association between meglitinide use and ND. In one study, meglitinide showed a significant protective effect against dementia in combination therapy rather than in monotherapy [43]. Because meglitinide is often used in combination with agents such as metformin rather than as a monotherapy, and in patients with diabetes who are not glycemically controlled despite multidrug therapy, there may be more meglitinide users among those who develop ND due to hyperglycemia [44,45]. However, the number of meglitinide users was too low to confirm this association.

Given that ARBs and CCBs are the first and second most prescribed drugs for hypertension in Korea as monotherapy, and the combination of ACEi/ARBs and CCBs is the first most prescribed drug in 2-drug therapy [46], CCBs are ranked higher in feature importance for the development of ND than ARBs. The preventive effect of CCBs on ND has been recognized in epidemiologic studies [47], and it is known that specific calcium channel subtypes are implicated in the pathogenesis of PD and that dihydropyridine CCBs with selectivity for these ion channels have a neuroprotective effect in animal models [48]. Although some conflicting studies have shown that antihypertensive drugs are not associated with ND [49], the results of this study show that CCB is effective in preventing ND.

Aspirin has previously been shown to reduce the incidence of AD and PD, as well as cardiovascular events and cancer. Aspirin-medicated acetylation prevents several neurodegenerative pathologies by interfering with protein aggregation [50]. Cilostazol has been shown to have a neuroprotective effect against vascular dementia in mice induced by L-methionine [51]. It is unclear whether the protective effects of aspirin and cilostazol against ND are due to an indirect lowering of the incidence of ND because of their pre-existing effects on reducing the risk of cardiovascular disease, another risk factor for ND, or whether they directly affect the pathological mechanisms of ND.

This study has several limitations. First, due to the retrospective nature of the study, obtaining accurate information from a dataset based on hospital medical records was difficult. Missing values, privacy regulations, and historical biases affect data availability for model training. The model was trained on data obtained from tertiary care centers, which may introduce selection bias because the patients may have different socioeconomic backgrounds from those of primary care patients and receive more comprehensive health care, thereby affecting ND risk and diabetes management. Information bias arising from inaccuracies in data-acquisition methods and the recording of medications and clinical parameters can result in the misclassification of both exposure and outcome, thus affecting the accuracy of the ML model’s predictions for ND in patients with T2DM. Second, the performance of this prediction model was not compared with that of other existing prediction models for ND in T2DM, and we expect that future comparative analyses will provide insights into the added value of the ML approach. Additionally, this study was limited to a prediction period of 3 years because of the availability and robustness of the follow-up data in the cohort. In the future, including data from longer periods (eg, 5 or 10 years) would strengthen the generalizability of the model for assessing long-term ND risk.

Furthermore, this study, on its own, cannot prove a causal relationship between the predictors used in the model and the incidence of ND. Confounding factors in predicting ND in patients with T2DM include possible biases due to medications prescribed for conditions such as hypertension or dyslipidemia; the effects of unexplained variables such as the severity and duration of diabetes; and missing data pertaining to lifestyle factors (smoking status, physical activity, diet, and alcohol consumption), all of which can distort the true relationship between the risk factors and ND outcomes. Further experimental research is needed to clarify the biological pathways and demonstrate the mechanisms of interaction between variables related to ND and their impact on the development of ND. Additionally, the dataset used was derived from hospital data based on *ICD-10* codes for PD and dementia only, which we grouped and defined as ND. Subcategories based on the pathology of ND were not considered, and other rare diseases such as multiple sclerosis, Huntington disease, and amyotrophic lateral sclerosis were not included in the ND outcome.

Also, integrating new models into existing health care systems can be challenging owing to compatibility issues with legacy software, potential disruption in clinical workflows, staff resistance, and the logistical and financial burdens of training. Hence, compatible software, user-friendly interfaces, and comprehensive training and support for health care professionals are necessary. Finally, the ML model used, which was trained and validated on data from Korean patients, may have limited generalizability to diverse populations owing to the different genetic, lifestyle, and environmental backgrounds. To enhance the applicability of the model, tone must incorporate data from diverse demographic groups and conduct external validation across different geographic locations and health care settings.

In conclusion, this study developed an ML-based prediction model using a representative national cohort. The model accurately predicted the risk of ND in all members of the Korean population with T2DM. We also demonstrate that the performance of several ML models is satisfactory. The AdaBoost model performed the best (AUROC 0.82 in the discovery dataset and AUROC 0.83 in the validation dataset). Our predictive model suggests that clinicians should consider age and cardiovascular disease, among other relevant variables, when assessing the risk of ND in patients with T2DM. This emphasizes the importance of comprehensive cardiovascular care and early intervention strategies to mitigate the risk of ND development in older patients with T2DM. This study is the first to apply an ML-based ND-prediction system to a national population with diabetes. In clinical practice, ML models enhance the prediction and management of ND by facilitating early intervention, personalizing treatment, optimizing resource allocation, and improving diagnostic accuracy. These models, when integrated with conventional diagnostics, can facilitate care for high-risk individuals and reduce long-term health care costs. The prediction model proposed in this study is expected to be competitive and cost-effective in preventing ND in Korean patients with T2DM and is expected to be widely used, especially in primary care settings. Future studies should focus on refining these models via longitudinal studies across diverse settings to address ethical concerns regarding data privacy and to promote multidisciplinary collaboration for advancing ND-prediction and treatment strategies.

## Appendix

Multimedia Appendix 1 Odds ratios with 95% CIs for neurodegenerative diseases in relation to medication use.

## Abbreviations

ACEi: angiotensin-converting enzyme inhibitor
AD: Alzheimer disease
ALP: alkaline phosphatase
ALT: alanine aminotransferase
ARB: angiotensin II receptor blocker
AST: aspartate aminotransferase
AUROC: area under the receiver operating characteristic curve
CCB: calcium channel blocker
GGT: gamma-glutamyl transferase
HbA1c: glycated hemoglobin
HDL: high-density lipoprotein
ICD-10: International Statistical Classification of Diseases, Tenth Revision
LDL: low-density lipoprotein
ML: machine learning
ND: neurodegenerative disease
OR: odds ratio
PD: Parkinson disease
ROC: receiver operating characteristic
T2DM: type 2 diabetes mellitus
TRIPOD: Transparent Reporting of a Multivariate Prediction Model for Individual Prognosis or Diagnosis

## Data Availability

The datasets generated and analyzed during the current study are not publicly available to protect patient privacy but are available from the corresponding author on reasonable request.
