# Supplementary material for: Machine Learning–Based Prediction of Neurodegenerative Disease in Patients With Type 2 Diabetes by Derivation and Validation in 2 Independent Korean Cohorts: Model Development and Validation Study
Source: J Med Internet Res. 2024 Oct 3;26:e56922. doi: 10.2196/56922 (PMC11487204; doi:10.2196/56922)
Supplement: Multimedia Appendix 1 [file jmir_v26i1e56922_app1.docx]

**Multimedia Appendix 1.** Odds ratios with 95% CIs for neurodegenerative diseases in relation to medication use.

| **Medication** | **Odds ratio (95% CI)** |
| --- | --- |
| Diabetes mellitus |  |
| Metformin | 1.01 (0.72 to 1.42) |
| Sulfonylurea | 0.72 (0.51 to 1.01) |
| DPP-4 inhibitor | 1.25 (0.82 to 1.91) |
| Meglitinide | 0.42 (0.24 to 0.75)^a^ |
| Thiazolidinedione | 0.72 (0.39 to 1.34) |
| α-Glucosidase inhibitor | 1.00 (0.44 to 2.27) |
| Insulin | 0.72 (0.51 to 1.01) |
| GLP-1 receptor agonist | N/A |
| SGLT2 inhibitor | 1.27 (0.40 to 4.00) |
| Hypertension |  |
| Angiotensin Ⅱ receptor blocker | 0.89 (0.63 to 1.25) |
| ACE inhibitor | 0.94 (0.50 to 1.74) |
| Calcium channel blocker | 0.93 (0.66 to 1.30) |
| Diuretics | 0.75 (0.53 to 1.07) |
| Beta blocker | 0.71 (0.50 to 1.02) |
| Dyslipidemia |  |
| Statin | 0.72 (0.51 to 1.03) |
| Fibrate | 1.35 (0.50 to 3.66) |
| Ezetimibe | 0.82 (0.44 to 1.53) |
| Antiplatelet |  |
| Aspirin | 0.59 (0.42 to 0.82)^a^ |
| Clopidogrel | 0.74 (0.52 to 1.05) |
| Cilostazol | 0.59 (0.38 to 0.92)^a^ |
| Glycoprotein IIb/IIIa antagonist | 0.55 (0.17 to 1.73) |

CI, confidence interval; DPP-4, dipeptidyl peptidase-4; GLP-1, glucagon-like peptide-1; SGLT2, sodium-glucose cotransporter-2; ACE, angiotensin-converting enzyme.

^a^This indicates a statistically significant difference based on logistic regression analysis (P <0.05).
